# Letter to the Editor regarding “Testotoxicosis: Report of Two Cases, One with a Novel Mutation in Luteinizing Hormone/Choriogonadotropin Receptor Gene”

**DOI:** 10.4274/jcrpe.2461

**Published:** 2015-12-03

**Authors:** Doğa Türkkahraman

**Affiliations:** 1 Antalya Education and Research Hospital, Clinic of Pediatric Endocrinology, Antalya, Turkey

**Keywords:** Testotoxicosis, mutation, LHCGR gene

## TO THE EDITOR

I read with great interest a paper recently published in JCRPE (2015) by Özcabı et al (1) entitled “Testotoxicosis: Report of Two Cases, One with a Novel Mutation in LHCGR Gene”. The authors report two cases with testotoxicosis caused by activating mutations in luteinizing hormone/choriogonadotropin receptor (LHCGR) gene. I thank the authors for this nice case report. However, there is a major point of concern regarding the follow-up criteria in these patients that I would like to highlight. In both cases, authors preferred to start up the treatment with a combination of bicalutamide and anastrozole. Then, on the follow-up of both cases, authors stated that because of insufficient suppression of pubertal progression and serum testosterone levels, bicalutamide was changed to ketoconazole, then normal serum level of testosterone was achieved with a combination of ketoconazole and anastrozole. Throughout the text, authors underline many times that as if the decline of serum testosterone is one of the main targets of the bicalutamide and anastrozole regimen.

Bicalutamide is a non-steroidal anti-androgen with highly selective competitive antagonistic activity to the androgen receptor (AR). Blockade of the AR by bicalutamide in the hypothalamus and pituitary gland suppresses the negative feedback of androgens on the release of LH. This, in turn, leads to a significant increase in androgen and estrogen levels (2). On the other hand, anastrozole is a third-generation aromatase inhibitor and blocks the conversion of androgens to estrogens leading to low estrogen, and in turn high androgen levels by the same mechanism described above (3). However, ketoconazole is an inhibitor of the steroidogenic enzyme CYP17A1 and blocks adrenal and testicular synthesis of testosterone at high doses. As understood from the mechanisms of action of these drugs, bicalutamide and anastrozole were not supposed to cause decrease in serum testosterone levels. However, ketoconazole can effectively decrease serum testosterone levels at high doses.

In a phase II, open-label pilot study by Reiter et al (4) including 14 male (2-9 years) with testotoxicosis, a largest cohort in the literature, it has been shown that bicalutamide and anastrozole combination in the treatment of testotoxicosis is safe and effective in reducing growth velocity and skeletal maturation. However, in these patients serum testosterone levels, signs and symptoms of virilization, and testicular volumes increased gradually during the study period.

In conclusion, as expected from the pharmacodynamics of the both drugs, bicalutamide and anastrozole can lead to increase in serum testosterone levels. Therefore, serum testosterone level should not be considered as a follow-up criterion in patients with testotoxicosis treated with bicalutamide and anastrozole combination. Instead, both growth velocity and skeletal maturation can be used as indicators in assessing the effectiveness of the combination therapy.
